# Measurement Properties of the European Portuguese Michigan Hand Outcomes Questionnaire (MHQ) in Paper and Electronic Formats: Instrument Validation Study

**DOI:** 10.2196/83563

**Published:** 2026-09-30

**Authors:** Eugénio Moita Gonçalves, Maria Catarina Silva, Henrique Ribeiro, Mariana Almeida, Bernardo Filipe, Sandra Branco, Filipa Henriques, Lilibeth Campos, Ana Campolargo, Pedro Cantista, Miguel Bernardes, Cristina Jácome

**Affiliations:** 1 Physical Medicine and Rehabilitation Department, Unidade Local de Saúde de Gaia e Espinho, Porto, Portugal Porto Portugal; 2 RISE-Health, Department of Community Medicine, Information and Health Decision Sciences, Faculty of Medicine of University of Porto, Porto, Portugal Porto, Porto Portugal; 3 Physical Medicine and Rehabilitation Department, Hospital da Prelada Porto Portugal; 4 Clinical Academic Center Egas Moniz Health Alliance Aveiro Portugal; 5 Physical Medicine and Rehabilitation Department, Unidade Local de Saúde de Santo António Porto Portugal; 6 Rheumatology Department, Unidade Local de Saúde de São João Porto Portugal; 7 Department of Medicine, Faculty of Medicine of University of Porto Porto Portugal

**Keywords:** Michigan Hand Outcomes Questionnaire, patient-reported outcome measures, digital technology, rehabilitation, psychometrics

## Abstract

**Background:**

The Michigan Hand Outcomes Questionnaire (MHQ) is a key instrument for assessing hand and wrist conditions, but a validated version for European Portuguese speakers is unavailable. Furthermore, the potential of an electronic format to improve data collection and clinical workflows has not been formally evaluated.

**Objective:**

This study aimed to translate and culturally adapt the MHQ for European Portuguese and to evaluate the measurement properties and comparability of its paper and electronic formats.

**Methods:**

An observational study was conducted in three phases: (1) translation and expert review, (2) content validation through cognitive interviews, and (3) psychometric evaluation in a cohort of 81 adult patients with hand or wrist conditions. Exploratory known-groups analyses were performed according to clinical, sociodemographic, and digital-access variables. Analysis also included internal consistency (Cronbach α and McDonald ω), measurement error (standard error of measurement [SEM]), relative and absolute reliability (intraclass correlation coefficient [ICC], Spearman correlation coefficient [*ρ*], and Bland-Altman analysis). The electronic format’s usability was assessed with the System Usability Scale (SUS).

**Results:**

The translation and cognitive interviews confirmed the content validity and conceptual equivalence of the European Portuguese MHQ. The instrument demonstrated excellent internal consistency (Cronbach α=0.85-0.98; McDonald ω=0.88-0.99). Exploratory known-groups analyses showed significant gender-related differences and suggested side-specific clinical discrimination in fracture cases. While group-level relative reliability between formats was strong (*ρ*=0.68-0.91; ICC=0.76-0.95), individual-level absolute reliability was poor, with wide 95% limits of agreement spanning a 32.83-point interval. Format-related score discrepancies were most pronounced among older participants, those with lower educational attainment, and those with low digital literacy. The electronic MHQ was the preferred format (43 out of 74, 58%), showed acceptable usability (median SUS score 70, IQR 66-88), and had lower SEM in pain and function domains.

**Conclusions:**

The European Portuguese MHQ is a relevant and reliable instrument with adequate measurement properties for both clinical practice and research. The electronic version demonstrated superior precision and user preference. However, maintaining the paper format as a viable alternative is essential to ensure equitable access for patients with digital barriers. Due to the overall low absolute reliability between formats, once a patient-preferred format is adopted, it should be used consistently for longitudinal patient monitoring.

## Introduction

Hand conditions, whether acute or chronic, are impactful due to the essential role of the hand in activities of daily living (ADL) and skilled tasks [1]. Acute hand injuries account for approximately 30% of emergency department visits, with common injuries including tendon damage, fractures, degloving injuries, and amputations [2]. The global incidence rate of hand and wrist fractures is 179 per 100,000 people, and, in Portugal, it is significantly higher at 271.2 per 100,000 inhabitants [3]. In addition, a burn center study revealed that 41.5% of burn victims had hand injuries, often resulting in long-term functional limitations [4].

Beyond acute trauma, the hand is also a primary site for manifestations of systemic and chronic conditions. For instance, rheumatoid arthritis may affect the hand and wrist, with a reported impairment prevalence of 94% [5,6]. Other prevalent hand conditions include osteoarthritis, DeQuervain’s tenosynovitis, carpal tunnel syndrome, trigger finger, and Dupuytren’s contracture, each of which can also significantly impair hand function [7]. Given the hand’s essential role in ADL and its high susceptibility to injury, clinical assessment must integrate physical examination findings with patient-reported outcomes. This dual approach prioritizes patient-centered functional goals and guides treatment strategies [4,8].

There is a growing emphasis on value-based, patient-centered care, where patient-reported outcome measures (PROMs) complement physical assessments. By capturing patients’ functional abilities, symptoms, and quality of life, PROMs enhance shared clinical decision-making, treatment monitoring, patient adherence, and overall care effectiveness and experience [9-13]. The Michigan Hand Outcomes Questionnaire (MHQ) is a widely recognized PROM for its comprehensive evaluation of hand and wrist conditions, assessing function, pain, ADL, work performance, hand appearance, and patient satisfaction. Notably, it is the only hand assessment PROM that effectively integrates components of the International Classification of Functioning, Disability, and Health framework, making it a valuable resource for complete patient evaluation [14,15]. The MHQ has also robust measurement properties, including minimal clinically important difference (MCID) values, which range from 8 to 13, with subscale-specific values varying depending on the diagnosis and treatment strategy [16,17]. It is often used in clinical research and included in standardized outcome sets, having received provisional endorsement from Outcome Measures in Rheumatology (OMERACT) and adoption by the International Consortium for Health Outcomes Measurement (ICHOM) for hand and wrist conditions [18,19].

Despite being validated in multiple languages, a European Portuguese version is still needed. Such an adaptation would ensure cultural and linguistic accuracy for the Portuguese population, enable comprehensive assessment of hand outcomes following conservative or surgical treatment in health settings, and support participation in international multicenter research [14,20,21].

With the increasing integration of digital technology into daily life and clinical practice, there is an opportunity for the implementation of electronic PROMs [22,23]. To the best of our knowledge, an electronic format of MHQ has not yet been published in peer-reviewed literature. This format could overcome the limitations of a paper version [20,24], support MHQ integration into clinical workflows, and improve patient engagement.

Thus, our study aimed to translate, culturally adapt, and validate the MHQ for European Portuguese, comparing its paper and electronic formats while evaluating its measurement properties and comparability.

## Methods

### Study Design

An observational study was conducted between February and December 2024 at the Physical Medicine and Rehabilitation (PMR) Department of the Unidade Local de Saúde de Gaia e Espinho. The study was divided into three phases: phase 1 – translation and expert panel review, phase 2 – content validity through cognitive debriefing, and phase 3 – measurement properties and comparability between formats. The study design followed the research principles outlined in the Declaration of Helsinki, with detailed procedures described in the Ethical Considerations section [25]. The study was reported according to the STROBE (Strengthening the Reporting of Observational Studies in Epidemiology) guidelines [26].

### Participants

Patients were enrolled only in phases 2 and 3 and were recruited by convenience sampling at our department. Eligible patients were aged 18 years or older, proficient in European Portuguese, and diagnosed with any hand and wrist conditions with functional impairment. We excluded participants who presented with hand conditions secondary to central nervous system disorders, as well as those with severe cognitive or visual impairments.

### Phase 1: MHQ Translation and Expert Panel Review

The MHQ is a comprehensive PROM assessing each hand using 37 core items across six subscales, I to VI, respectively: I - overall hand function (5 items per hand; eg, “Overall, how well did your right hand work?”), II - ADL (5 items per hand and 7 for two-handed activities; eg, “Carry grocery bags”), III - work performance (5 items; eg, “How often were you unable to do your work because of problems with your hand(s)/wrist(s)?”), IV - pain (5 items per hand; eg, “How often did the pain in your right hand/wrist interfere with your sleep?”), V - appearance (4 items per hand; eg, “I am satisfied with the appearance (look) of my right hand”), and VI - patient satisfaction with hand function (6 items per hand; eg, “Motion of the fingers in your right hand”) [27]. Except for work performance, all subscales are evaluated separately for each hand [14,15]. Items are scored on a 1-5 Likert scale, summed, and converted to a 0-100 scale. Higher subscale scores indicate better outcomes, except for pain.

For unilateral conditions, the score of the affected hand is used. For bilateral conditions (eg, rheumatoid arthritis), the right- and left-hand scores are averaged. The total score is the average of all subscales, with the pain score reversed. At the end of the MHQ, a set of questions proposed by the original authors collects demographic information, including dominant hand, ethnicity, job-related details, gender, education level, and annual family income. The questionnaire takes approximately 15 minutes to complete and can be self-administered by patients or aided by health care professionals [20].

A license for academic research was obtained from the MHQ copyright holders to translate the questionnaire into European Portuguese and to develop an electronic format. Following international guidelines, the translation and cross-cultural adaptation process began with two independent translations conducted by native Portuguese-speaking PMR residents fluent in English (EMG and MA) [28,29]. The socioeconomic questions of the sociodemographic section were also adapted to ensure cultural relevance. The 2 translations were merged and disagreements identified. Both translations were reviewed item by item during an online meeting with a panel of experts. The expert panel included the 2 translators, a PMR physician (PC), a rheumatologist (MB), and a PROM expert (CJ). A synthesis of the discussion produced a preliminary Portuguese version. In accordance with the Beaton et al [28] methodology, the preliminary version was back-translated into English by two translators: a native Canadian English speaker and a native Portuguese speaker with professional fluency in English from over 25 years of living and working in English-speaking countries. Both back-translators were non–health care professionals and were unaware of the original MHQ. The expert panel reviewed and resolved discrepancies between the two back-translated versions. The final European Portuguese MHQ and the back-translated version were approved by the original authors.

### Phase 2: Content Validity Through Cognitive Debriefing

Cognitive debriefing interviews were conducted with patients with hand conditions, following established qualitative research guidelines and the Consensus-based Standards for the selection of health Measurement Instruments (COSMIN) recommendations [30,31]. A structured interview guide was used, incorporating think-aloud techniques and probing questions to assess the MHQ relevance, comprehensiveness, and comprehensibility.

The interviews were conducted by two trained PMR residents (EMG or BF); both received specific training in cognitive debriefing techniques prior to the study to ensure procedural consistency. Participants were encouraged to clarify and confirm their interpretations, ensuring accurate data collection. The interviews were audio-recorded, and field notes were taken to capture nonverbal cues. Verbatim transcriptions were thematically analyzed by two researchers (EMG, male, and CS, female) to identify ambiguities and key issues [31]. An initial sample size of 8 to 10 patients was planned, but data collection continued until thematic saturation was reached. This was defined as the point at which no new themes or insights emerged in 2 subsequent interviews, and responses became redundant, occurring at the 10^th^ interview [32].

Participants also reported their clinical diagnosis and provided sociodemographic information, including age, ethnicity, gender, educational level, annual family income, and dominant hand.

Following cognitive debriefing, patient feedback was reviewed by the expert panel to determine whether revisions were required. Suggested changes were considered only when they improved clarity without compromising the original construct. Final decisions were made by consensus after reviewing the interview findings and the corresponding original item content.

### Phase 3: Measurement Properties and Comparability Between Formats

In line with COSMIN recommendations, at least 50 participants were recruited and randomly assigned to complete either the paper or electronic format first [29,33]. To mitigate recall bias, participants completed the alternate format within one week of the initial MHQ administration to ensure consistency in their reported clinical and functional status [34].

The electronic format was developed in accordance with the International Society for Pharmacoeconomics and Outcomes Research (ISPOR) best practices [33,35]. The migration from the paper format involved specific design choices to optimize user experience while adhering to specific migration principles. The visual layout was modified from the paper grid layout to a vertical list in the electronic format, where each item was followed by its corresponding radio-button response options. All items belonging to a single subscale were presented together on one screen, a design intended to maintain context for the participant. The instrument’s skip logic for the pain subscale was preserved using conditional logic; if a participant selected “Never” (*Nunca*) in response to the initial pain frequency question, the subsequent branched questions about pain characteristics were automatically hidden and could not be answered. The format is hosted on a secure, web-based platform (Jotform) compliant with the European General Data Protection Regulation (GDPR). It was designed to ensure accessibility, provide real-time scoring for each MHQ domain and the total score, and enable efficient data analysis. Indeed, upon completing the electronic format on an 11-inch tablet device, patients were redirected to an interface that uses OpenAI’s ChatGPT-4o, an AI tool, to deliver personalized MHQ results with a report. This report includes comparisons to normative data from US patients with and without hand conditions [36-38]. A demonstration video is available in Multimedia Appendix 1.

After completing the electronic format, participants assessed its usability using the European Portuguese version of the System Usability Scale (SUS) [39]. The SUS is a standard 10-item questionnaire that uses alternating positive and negative statements to avoid response bias. Examples of positive items include “I think that I would like to use this system frequently” and “I thought the system was easy to use,” while negative items include statements such as “I found the system unnecessarily complex.” SUS scores range from 0 to 100, with scores of 68 or above indicating acceptable [39]. Participants also indicated their format preference: paper, electronic, or no preference.

Participants also provided clinical and sociodemographic data, as described in phase 2, and reported their digital literacy. Digital literacy was evaluated using the text messaging and smartphone usage subscales from the validated European Portuguese version of the Media and Technology Usage and Attitudes Scale (MTUAS) [40,41]. This assessment consisted of 11 questions assessing activities such as sending and receiving text messages, browsing the web, and using mobile apps. Each item is rated on a 10-point Likert scale (1=never to 10=all the time), reflecting the frequency of use. The digital literacy score was determined by calculating the average of all item scores, yielding a final score ranging from 1 to 10.

### Data Analysis

Participants’ characteristics in phases 2 and 3, as well as MHQ scores, were described using descriptive statistics. Normality was assessed through the Kolmogorov-Smirnov test and histogram inspection. Normally distributed data were presented as mean (SD), while nonnormally distributed data were summarized using median and quartiles (Q1 and Q3).

Missing data were handled according to their type. Structural missing data, which arose from the MHQ’s intended skip-pattern logic in the pain subscale (IV-A and IV-B), were preserved as valid nonresponses. Little’s test indicated that sporadic missing data were not missing completely at random. Therefore, multiple imputation using the predictive mean matching method was performed [42].

Exploratory known-groups analyses were conducted to examine whether affected-hand MHQ scores differed across clinical (dominant-hand involvement), sociodemographic (age group, income level, educational attainment, and gender), and digital-access (digital literacy and usability) subgroups. Between-group comparisons were performed using Mann-Whitney *U* tests. To further assess comparability between administration formats, paired comparisons between paper and electronic scores were performed within each subgroup using Wilcoxon signed-rank tests.

Internal consistency was assessed using Cronbach ⍺ (α>0.70 considered acceptable), and McDonald ω as a more robust measure [34,43].

Comparability between formats was evaluated using several methods. At the group level, relative reliability was assessed using the intraclass correlation coefficient (ICC) based on a 2-way random-effects, single-rater, absolute agreement model, with values interpreted as poor (<0.50), moderate (0.50-0.74), good (0.75-0.89), or excellent (≥0.90); and Spearman rank correlation coefficient (*ρ*) for association between format scores [44]. For individual-level agreement, the analysis of absolute reliability was conducted to visualize systematic bias and quantify random error, using the Bland-Altman method by calculating the mean difference and 95% limits of agreement (LoA) and through standard error of measurement (SEM), calculated separately for each format to estimate the margin of error for an individual’s score. Participants who completed only one format were excluded from these analyses.

Format preferences were explored according to gender, age group, educational attainment, and digital literacy using Mann-Whitney *U* tests.

Statistical analyses were performed using IBM SPSS Statistics (version 29.0) and R (version 4.2.0), with a *P* value less than .05 considered statistically significant.

### Ethical Considerations

This study was conducted in accordance with the principles of the Declaration of Helsinki [25]. Ethical approval was granted by the Health Ethics Committee of the Unidade Local de Saúde de Gaia e Espinho (reference number 62/2023, addendum numbers 64/2024-1 and 187/2024). All participants received clear explanations of the study objectives and provided written informed consent prior to any data collection. To protect participant privacy and confidentiality, all collected data were strictly deidentified and stored securely. Participants did not receive any financial compensation for their involvement in this study.

## Results

### Phase 1: Translation and Expert Panel Review

The proposed adaptation maintains the original MHQ context while incorporating linguistic and cultural refinements to ensure clarity and accessibility. These modifications, informed by expert panel discussions, enhanced the questionnaire’s relevance for Portuguese-speaking users, regardless of their educational background. For example, “gestures” (*gestos*) was chosen over “activities” (*atividades*) to better capture the nature of hand movements required for the tasks described in the questionnaire items related to the left and right hand, respectively. Similarly, a few response options were modified to reflect expressions commonly used in Portuguese, for instance, “very poor” (*muito pobre*) to “very bad” (*muito mal*). Additionally, the term “normal work” (*trabalho normal*) was translated as “usual work” (*trabalho habitual*), as it more accurately conveys the concept of routine professional and daily tasks in Portuguese. Some English terms also lacked a direct Portuguese equivalent that would be appropriate in the given context. For instance, “sensation (feeling)” was translated as “sensitivity to touch” (*sensibilidade [ao toque]*), since the literal Portuguese equivalent of “feeling” (*sentimento*) is primarily associated with emotions rather than tactile perception. Similarly, “look” was translated as “aspect” (*aspecto*), as the word “look” (*olhar*) in Portuguese refers to the act of looking rather than physical appearance. Additionally, “pain level” (*nível de dor*) was translated as “pain intensity” (*intensidade da dor*), which is a more commonly known term among Portuguese-speaking patients. Adjustments were also made in the demographic section. Demographic categories, including ethnicity, gender, job-related questions, education, and income, were adapted to align with Portuguese cultural and statistical standards, using terms adopted from the Portuguese National Institute of Statistics.

### Phase 2: Content Validity Through Cognitive Debriefing

A total of 12 patients participated in the cognitive debriefing phase (Table 1 and Figure 1), and interviews lasted between 20 and 40 minutes. Participants had a median age of 54, IQR 49-57 years, were predominantly female (8 out of 12, 67%), and mostly non-Hispanic White (11 out of 12, 92%). Most patients demonstrated a clear understanding of the questionnaire's content. However, adjustments were needed based on participants’ suggestions. As highlighted in Table 2, some expressions raised doubts regarding interpretation. Regarding finger movement (subscales I and VI), 2 patients asked for help before answering, as not all fingers were equally affected by stiffness and immobility. The same issue applied to the term “sensibility,” where patients were unsure which specific finger they should base their answer on, given that sensory perception varies across different digits. In subscale V, several patients noted that the response system was not as intuitive as in previous subscales, considering that the items in these tables were in the affirmative form and not in the interrogative form.

All findings were discussed with the expert panel. Because these are common challenges when completing standardized questionnaires, and a primary goal was to preserve the structural integrity of the original instrument as much as possible to ensure comparability, modifications were kept subtle and limited to minor wording adjustments, with no changes made to the original MHQ structure.

**Table 1:** Patients’ sociodemographic and clinical data (N=12).

Variables			Values						
Age (years), median (IQR)			54 (49-57)						
Ethnicity, n (%)									
	Non-Hispanic White	11 (91.7)							
	Mixed origin or heritage	1 (8.3)							
Sex, n (%)									
	Female				8 (66.7)				
	Male							4 (33.3)	
Education level, n (%)									
	Primary education (1^st^ cycle)				2 (16.7)				
	Secondary education				5 (41.7)				
	Bachelor’s degree				1 (8.3)				
	Licentiate degree				4 (33.3)				
Annual family income (in Euros)^a^, n (%)									
	0 to 5000						4 (33.3)		
	5001 to 10,000						2 (16.7)		
	19,001 or above						5 (41.7)		
	Prefer not to answer						1 (8.3)		
Dominant hand, n (%)									
	Right-handed								12 (100)
Disability laterality, n (%)									
	Right					6 (50)			
	Left					2 (16.7)			
	Bilateral					4 (33.3)			
Diagnostic group, n (%)									
	Fractures and dislocations					5 (41.7)			
	Peripheral neuropathies					2 (16.7)			
	Systemic disease					3 (25)			
	Degenerative joint disease					1 (8.3)			
	Muscle and/or tendon injury					1 (8.3)			
Diagnosis, n (%)									
	Distal radius fracture				3 (25)				
	Carpal tunnel syndrome				2 (16.7)				
	Metacarpal fracture			1 (8.3)					
	Rheumatoid arthritis			2 (16.7)					
	Carpal bone fracture			1 (8.3)					
	Tenosynovitis			1 (8.3)					
	Systemic sclerosis			1 (8.3)					
	Hand osteoarthritis			1 (8.3)					

^a^The average exchange rate at the time of data collection was 1 EUR=US $1.08.

**Figure 1:**
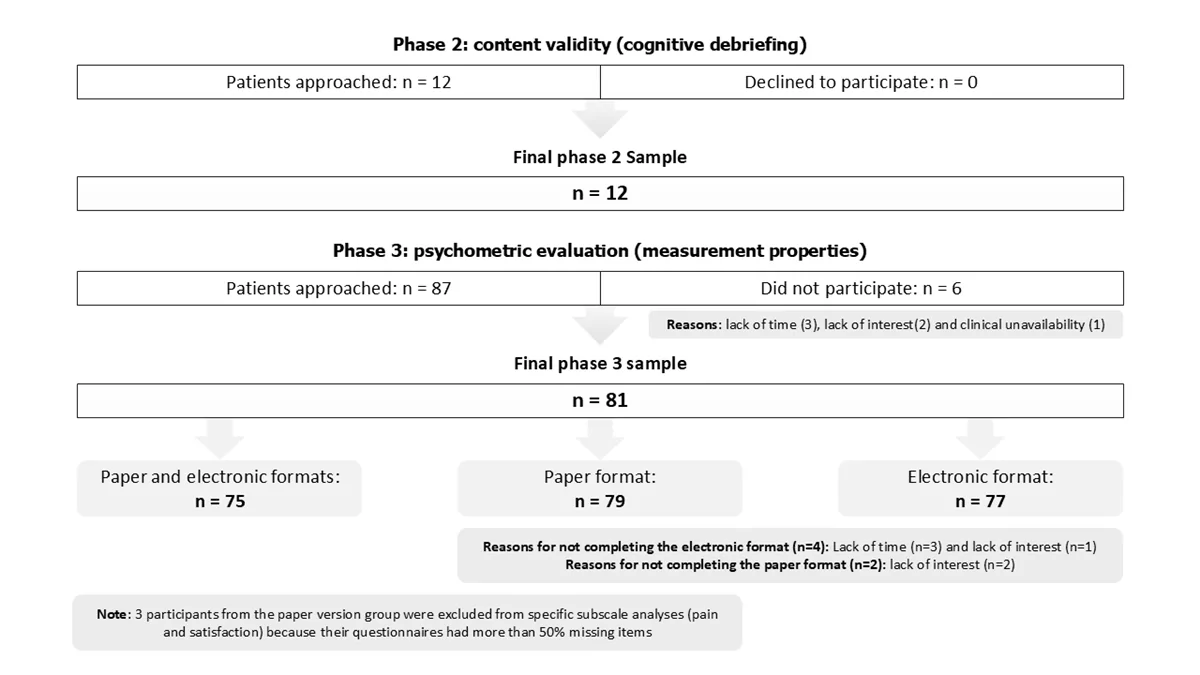
Flowchart outlines patient enrollment and retention across phase 2 (content validity) and phase 3 (psychometric evaluation). Phase 2 included all 12 approached patients. In phase 3, 81 of 87 approached patients participated, with reasons provided for the 6 nonparticipants. The final phase 3 sample (n=81) details Michigan Hand Outcomes Questionnaire format completion rates: 75 completed both formats, 79 completed the paper format, and 77 completed the electronic format. Additionally, the flowchart notes the exclusion of 3 participants from specific subscale analyses due to missing data.

**Table 2:** Examples of controversial expressions identified by patients during cognitive interviews.

MHQ^a^ subscale	Original expression	Rationale	Revised expression
Activities of Daily Living Item II^b^-C4	“Carry a grocery bag”	Although there is a direct translation in Portuguese for “carry a grocery bag” (*carregar um saco de mercearia*), this expression does not reflect the need to use both hands as it was intended by the original item. Since most patients usually carry a grocery bag in one hand only, we considered more appropriate to replace it with “carry grocery bags” (*carregar sacos de mercearia*) to translate a bilateral activity.	“Carry grocery bags” (*Carregar sacos de mercearia*)
Activities of Daily Living Item II-C5	“Wash dishes”	It was not considered a common chore. The alternative “loading the dishwasher” (*colocar a loiça na máquina*) was suggested to be added. The expert panel considered that would involve a different hand activity and could alter the construct assessed by the original item.	No revision was made
Work Performance Subscale III	—^c^	Items 3, 4 and 5 were perceived as identical. However, no patient suggested a more suitable alternative.	No revision was made
Pain Items IV-A2 and IV-B2	“Please describe the pain you had in your hand/wrist” (*Por favor, descreva a dor que teve na sua mão/punho*)	There were doubts about whether the statement referred to the maximum pain the patient felt or the average. Furthermore, it was not understandable if these items were related to the pain while at rest or during activity. As the identified ambiguity is also present in the original instrument, the item was intentionally left unchanged.	No revision was made
Pain Items IV-A5 and IV-B5	“Unhappy”	As this word carries a substantial meaning, some patients suggested the alternative “sad” (*triste*).	“Sad” (*triste*)
Satisfaction Items VI-A1 and VI-B1	“Overall function”	The translated expression “overall function” (*função global*) was unclear to some participants, particularly older patients, who did not understand its meaning. The alternative “general function” (*função geral*) was suggested by one participant, but the participant also understood the wording *“função global*.”	No revision was made

^a^Michigan Hand Outcomes Questionnaire.

^b^Roman numerals (I-VI) indicate Michigan Hand Outcomes Questionnaire (MHQ) subscales (eg, II=activities of daily living, IV=pain), letters denote the hand assessed (A=right, B=left, C=both), and numbers represent the question order within the subscale (eg, IV-A2=2nd Pain question for the right hand).

^c^Not applicable.

### Phase 3: Measurement Properties and Comparability Between Formats

#### Participants’ Characteristics

A total of 81 participants were included in this study phase. Their demographic and clinical characteristics are summarized in Table 3. The median age was 55 (45-67) years, with most participants being women (51 out of 81, 63%). Median digital literacy score was 5.45 (3-6.6), and almost half (39 out of 81, 48%) completed primary education. Right-hand dominance was high (76 out of 81, 94%), with disability affecting mostly the right (36 out of 81, 44%). Fractures and dislocations (42 out of 81, 52%), especially distal radius fractures (29 out of 81, 36%), were the most frequent conditions.

**Table 3:** Patients’ demographic and clinical data (N=81).

Variables			Values^a^
Age (years), median (Q1-Q3)			55 (45-67)
Digital literacy, median (Q1-Q3)^b^			5.4 (3-6.6)
Ethnicity			
	Non-Hispanic White	75 (92.6)	
	Non-Hispanic Black	2 (2.5)	
	Mixed origin or heritage	1 (1.2)	
	Prefer not to answer	1 (1.2)	
	Missing	2 (2.5)	
Sex, n (%)			
	Female	51 (63)	
	Male	28 (34.6)	
	Prefer not to answer	1 (1.2)	
	Missing	1 (1.2)	
Education level, n (%)			
	Primary education (first cycle)	20 (24.7)	
	Primary education (second cycle)	10 (12.3)	
	Primary education (third cycle)	9 (11.1)	
	Secondary education	19 (23.5)	
	Bachelor’s degree	2 (2.5)	
	Licentiate degree	12 (14.8)	
	Master’s degree	6 (7.4)	
	Missing	3 (3.7)	
Annual family income (in Euros)^c^, n (%)			
	0 to 5000	11 (13.6)	
	5001 to 10,000	8 (9.9)	
	13,501 to 19,000	12 (14.8)	
	19,001 to 27,500	9 (11.1)	
	Prefer not to answer	21 (25.9)	
	Others	16 (19.8)	
	Missing	4 (4.9)	
Dominant hand, n (%)			
	Right-handed	76 (93.8)	
	Ambidextrous	1 (1.2)	
	Missing	4 (4.9)	
Disability laterality, n (%)			
	Right	36 (44.4)	
	Left	33 (40.7)	
	Bilateral	12 (14.8)	
Diagnostic group^d^, n (%)			
	Fractures and dislocations	42 (51.9)	
	Peripheral neuropathies	12 (14.8)	
	Muscle and/or tendon injury	6 (7.4)	
	Systemic disease	8 (9.9)	
	Others	15 (18.5)	
Diagnosis^d^, n (%)			
	Distal radius fracture	29 (35.8)	
	Carpal tunnel syndrome	10 (12.3)	
	Metacarpal fracture	7 (8.6)	
	Rheumatoid arthritis	5 (6.2)	
	Carpal bone fracture	4 (4.9)	
	Tenosynovitis	4 (4.9)	
	Dupuytren’s disease	3 (3.7)	
	De-gloving injury	3 (3.7)	
	Others	24 (29.6)	

^a^Values shown as n (%) unless otherwise indicated; n: absolute frequency; %: relative frequency; Q1-Q3: IQR.

^b^Digital literacy was calculated using the Media and Technology Usage and Attitudes Scale (MTUAS), with scores ranging from 1 to 10 points.

^c^The average exchange rate at the time of data collection was 1 EUR=US $1.08.

^d^Total percentage exceeds 100% as participants could have more than one diagnosis.

Three participants’ paper questionnaires exhibited more than 50% of missing items in the pain (2) and satisfaction (1) subscales, which were excluded from specific subscale analysis. Isolated missing values were present in two paper questionnaires related to items II-B2 (“pick up a coin”) and VI-A5 (“pain level of your right hand”). No missing values were observed in the electronic MHQ, as fields were mandatory by design.

#### MHQ Scores and Known-Group Analyses

The distribution of scores for the paper and electronic formats was highly similar across nearly all subscales, with comparable median scores and IQRs, although subgroup analyses showed selected format-related differences (Table 4).

**Table 4:** Descriptive statistics of European Portuguese Michigan Hand Outcomes Questionnaire (MHQ) scores in both formats.

MHQ subscales	Paper format, median (IQR)	Electronic format, median (IQR)
Overall hand function of the right hand	70 (50-90)	70 (50-90)
Overall hand function of the left hand	75 (45-100)	70 (50-95)
ADL^a^ with the right hand	75 (50-100)	75 (55-100)
ADL with the left hand	75 (40-100)	75 (50-100)
ADL with both hands	60.7 (35.7-82.1)	57.1 (35.7-75)
Work performance	50 (35-70)	50 (25-67.5)
Pain in the right hand	22.5 (0-51.3)	25 (0-50)
Pain in the left hand	17.5 (0-45)	10 (0-45)
Appearance of the right hand	68.8 (37.5-100)	81.3 (50-96.9)
Appearance of the left hand	75 (31.3-100)	75 (43.8-100)
Satisfaction with the right hand	75 (45.8-92.7)	75 (39.6-100)
Satisfaction with the left hand	75 (36.5-100)	75 (39.6-100)
Total score of the right hand	68.9 (47.3-83.1)	69.8 (47.1-83.3)
Total score of the left hand	68.5 (48.6-80.9)	69.7 (49.5-84.1)
Total score of both hands	63.90 (53.7-77.4)	64 (54.4-76.1)

^a^ADL: activities of daily living.

Exploratory known-groups analyses showed a clinically interpretable pattern in the distal radius fracture subgroup. Dominant-hand involvement alone did not consistently differentiate MHQ scores. However, hand-specific analyses by fracture side indicated side-specific clinical discrimination, particularly in the paper format, where total scores were significantly lower for the fractured hand than for the contralateral hand in both right- and left-sided fractures. This pattern was only partially reproduced in the electronic format, where the left-hand total score remained significantly lower among participants with left-sided fractures. Contralateral, uninjured-hand scores remained below the maximum possible value at the group level in both formats.

Among sociodemographic subgroups, gender showed the most consistent differences across formats, with men reporting higher scores in several MHQ domains, including function, ADL, work performance, satisfaction, and total score. Education-related differences were observed mainly in the paper format, where participants with lower educational attainment reported lower work performance, appearance, and total scores. Format-related differences were more frequent among older participants, those with lower educational attainment, lower digital literacy, and lower perceived usability, particularly for appearance and total scores.

#### Internal Consistency

As shown in Table 5, internal consistency was good to excellent across all subscales as demonstrated by Cronbach α values ranging from 0.85 to 0.98, while McDonald ω values ranged from .88 to .99. The highest internal consistency was observed in the “Satisfaction with the left hand” subscale for both coefficients.

**Table 5:** Internal consistency of the European Portuguese Michigan Hand Outcomes Questionnaire (MHQ) and reliability between formats.

MHQ subscales	Cronbach α	McDonald ω	*ρ* ^a^	ICC^b^ (95% CI)	SEM^c^			
						Paper format		Electronic format
Overall hand function of the right hand	0.95	0.97	0.68	0.80 (0.68-0.87)	2.29		1.28	
Overall hand function of the left hand	0.96	0.97	0.89	0.93 (0.90-0.96)	2.31		1.12	
ADL with the right hand	0.95	0.96	0.76	0.85 (0.76-0.90)	1.72		1.75	
ADL with the left hand	0.96	0.98	0.91	0.95 (0.93-0.97)	2.71		2.97	
ADL with both hands	0.95	0.97	0.87	0.92 (0.87-0.95)	4.05		2.95	
Work performance	0.95	0.96	0.82	0.89 (0.83-0.93)	2.41		2.13	
Pain in the right hand	0.94	0.99	0.72	0.85 (0.76-0.90)	3.88		2.21	
Pain in the left hand	0.94	0.99	0.88	0.80 (0.69-0.88)	3.44		1.97	
Appearance of the right hand	0.86	0.89	0.76	0.80 (0.66-0.88)	1.35		1.39	
Appearance of the left hand	0.85	0.88	0.74	0.86 (0.78-0.91)	2.04		2.16	
Satisfaction with the right hand	0.95	0.98	0.71	0.76 (0.62-0.85)	3.45		2.76	
Satisfaction with the left hand	0.98	0.99	0.75	0.86 (0.78-0.91)	3.95		2.91	
Total score of the right hand	—^d^	—	0.85	0.81 (0.71-0.88)	—		—	
Total score of the left hand	—	—	0.89	0.91 (0.86-0.94)	—		—	
Total score of both hands	—	—	0.83	0.88 (0.82-0.93)	—		—	

^a^All Spearman rank correlation coefficients (*ρ*) were statistically significant (*P*<.01).

^b^ICC: intraclass correlation coefficient.

^c^SEM: standard error of measurement.

^d^Not applicable.

#### Reliability and Comparability Between Formats

The analysis of relative reliability between the paper and electronic formats revealed a good to excellent reliability, with ICC ranging from 0.76 to 0.95 (Table 5). These findings were supported by strong and statistically significant Spearman coefficients (*ρ*=0.68 to 0.91; *P*<.01).

In contrast, the absolute reliability analysis demonstrated slight measurement error at the individual level. While Bland-Altman analysis (Figures 2-4) confirmed negligible systematic bias, it revealed wide 95% LoA. For the total score of both hands, this interval spanned a substantial 32.83 points (from –18.26 to 14.57). Reinforcing the differences in measurement error, the analysis of the SEM showed the electronic version to be metrically superior. For instance, the SEM for pain in the right hand was 3.88 for the paper format compared to a much lower 2.21 for the electronic format (Table 5).

**Figure 2:**
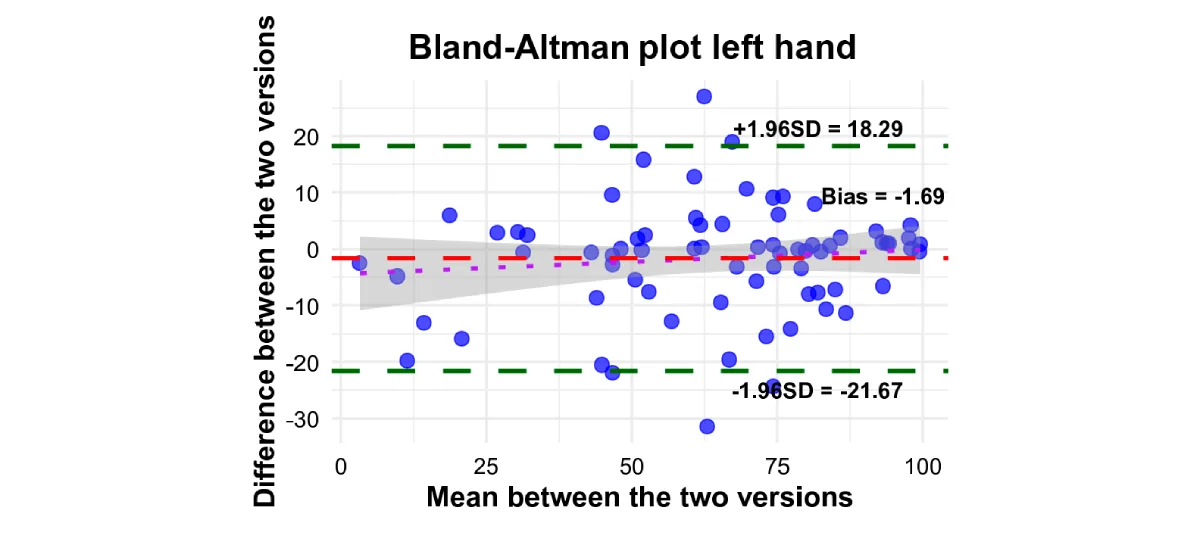
Bland-Altman plot comparing paper and electronic Michigan Hand Outcomes Questionnaire total score for the left hand. The mean difference is shown with 95% limits of agreement calculated as mean (SD 1.96).

**Figure 3:**
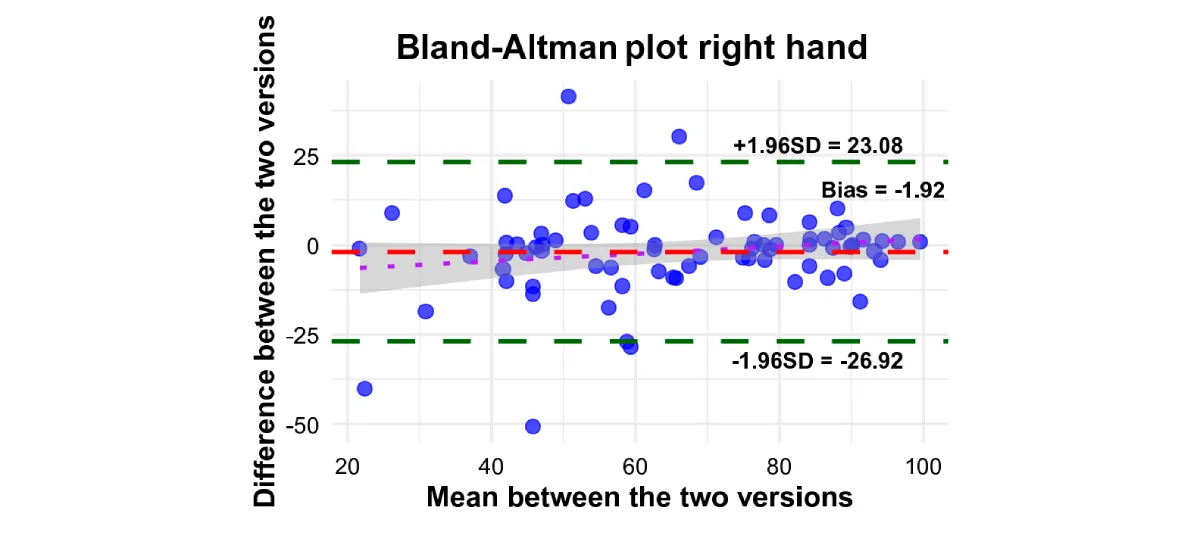
Bland-Altman plot comparing paper and electronic Michigan Hand Outcomes Questionnaire total score for the right hand. The mean difference is shown with 95% limits of agreement calculated as mean (SD 1.96).

**Figure 4:**
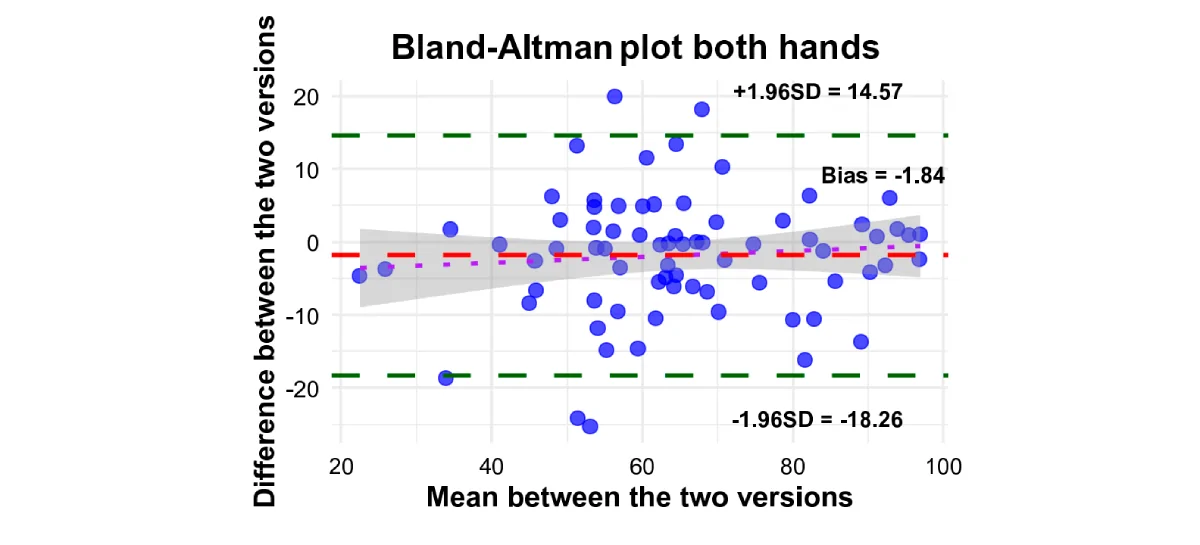
Bland-Altman plot comparing paper and electronic Michigan Hand Outcomes Questionnaire total score for both hands. The mean difference is shown with 95% limits of agreement calculated as mean (SD 1.96).

#### Administration Preference and Usability

Most participants (43 out of 74, 58%) preferred the electronic format, whereas 15 (20%) preferred the paper version, and 16 (22%) were indifferent. This preference was more pronounced among individuals with higher digital literacy (24 out of 36, 67%) and higher education background (13 out of 18, 72%). It was also favored by less frequent technology users (19 out of 38, 50%) and those with less formal education (30 out of 56, 54%). Notably, more than half (12 out of 22, 55%) of participants aged 65 and older also favored the electronic format, closely tracking the preference seen in younger participants (31 out of 52, 60%). Similarly, gender did not significantly influence preference, with the electronic format being the top choice for both male (18 out of 27, 67%) and female (25 out of 47, 53%) participants. Regarding usability, this format scored a median of 70 (66-88) on the SUS.

## Discussion

### Principal Findings

This study translated and culturally adapted the MHQ for European Portuguese and conducted an evaluation of its measurement properties in both paper and electronic formats. The adaptation process, informed by expert review and patient cognitive interviews, yielded an instrument that was well-understood and relevant. Exploratory known-groups analyses provided partial evidence of clinical relevance. The subsequent psychometric evaluation revealed high internal consistency across all subscales in both formats and an overall adequate relative and absolute reliability between the paper and electronic formats supporting their use in clinical practice and research.

### Content Validity and Clinical Relevance

The translation and cultural adaptation process confirmed the relevance and comprehensibility of the European Portuguese MHQ. Cognitive interviews showed that most participants understood the questionnaire, although some items generated uncertainty, particularly those involving abstract concepts such as “overall function,” hand appearance, and satisfaction. These findings are clinically important because they show that even when a PROM is linguistically accurate, some concepts may still be interpreted differently by patients depending on their condition, education, and lived experiences. Importantly, no structural changes were made to the original MHQ, preserving comparability with other validated versions [31].

Exploratory known-groups analyses provided evidence of clinical relevance. Gender showed consistent sociodemographic discrimination across both administration formats, with men generally reporting better function and lower pain burden than women. However, these findings should not be interpreted as reflecting a direct effect of gender alone, as they may also reflect differences in diagnosis, disease severity, occupational exposure, pain perception, treatment stage, or other unmeasured clinical factors [45].

In the distal-radius-fracture subgroup, affected-hand MHQ scores did not differ consistently according to dominance status, suggesting that dominance alone was not a major determinant of perceived disability. More clinically relevant was the observation that contralateral, uninjured-hand scores remained below the maximum possible score. This supports previous MHQ normative findings indicating that unilateral hand or wrist problems may also reduce perceived function of the unaffected hand, likely because many daily tasks require bilateral hand use or because impairment in one hand can increase demand on the other [46].

Therefore, contralateral scores after unilateral distal radius fracture should not necessarily be interpreted as fully “normal” and bilateral summary scores may dilute side-specific impairment. The absence of clear work-performance differences between right- and left-sided fracture groups further suggests that occupational impact may depend on factors beyond injury side or dominance alone, including task demands, compensatory strategies, pain, rehabilitation stage, and postinjury adaptation.

### Reliability and Comparability Between Formats

This study provides evidence for the comparability between paper and electronic formats. The high Spearman correlations (*ρ* up to 0.91) and excellent ICC values (up to 0.95) indicate that, on average, the two formats produce highly comparable scores. This finding supports data collection via either format within a single study. While the formats are equivalent for groups, the Bland-Altman analysis showed they might not be interchangeable for individuals. The 95% LoA for the total score of both hands spanned a 32.83-point interval. This is a substantial degree of random error when compared to the MHQ’s established MCID, which is 8 to 13 points [16]. For a patient, a score difference generated simply by switching from paper to electronic format could be 2 to 3 times larger than a genuine, clinically meaningful change. This finding strongly cautions against using the two formats interchangeably to monitor a single patient’s progress. Format-related differences were more likely to appear among participants with lower educational attainment, lower digital literacy, lower perceived usability, older age, and female gender. The inherently subjective appearance domain was the most consistently affected by administration format, and total score differed between formats in selected subgroups [47].

Furthermore, the analysis of measurement precision via SEM suggests that the electronic version is not merely an alternative but, in some respects, a metrically superior instrument. The electronic format demonstrated a significantly lower SEM for the crucial domains of hand function and pain, meaning it has less inherent measurement “noise” and can therefore detect smaller true changes more reliably than the paper format.

Given its precision, lack of missing data, and patient preference, the electronic format appears suitable as the preferred administration format in many settings. Nevertheless, maintaining a paper alternative remains advisable to ensure access for patients with lower digital familiarity, lower perceived usability, or specific technological barriers. For clinical care involving longitudinal assessments, the two formats should never be used interchangeably for the same patient.

### Strengths, Limitations, and Contribution to Practice

This study’s strengths include its adherence to COSMIN and ISPOR guidelines, the direct comparison between paper and electronic formats, and a mixed methods approach that allowed qualitative findings to explain quantitative results. The development of a preferred electronic version with acceptable usability is a step toward modernizing data collection in Portuguese rehabilitation settings [35,48]. Patient preference for electronic MHQ was strongly influenced by age, education, and digital literacy. While younger, highly educated patients showed the most preference, acceptance among older individuals points to a shrinking digital divide. These findings highlight the need for user-friendly electronic tools alongside traditional paper forms to guarantee accessibility [37].

Nonetheless, limitations must be acknowledged. The use of a convenience sample from a single center may limit generalizability. Structural validity was not examined because of the modest sample size and multidimensional structure of the MHQ. Convergent and divergent validity were also not formally assessed because no external comparator instrument was administered. Both paper and electronic formats were completed in the presence of a health care professional, allowing clarification when needed. However, assistance during completion was not systematically recorded. Based on clinical observation, assistance appeared more frequent with the paper format, possibly because writing, turning pages, and handling the questionnaire may be challenging for patients with hand or wrist functional impairment [49]. Therefore, the frequency and potential influence of assistance on responses could not be quantified. The electronic format was tested only on an 11-inch tablet in a controlled clinical setting, and usability may differ in unsupervised or device-diverse contexts. Although mandatory response fields prevented missing data, they may have introduced response bias by forcing uncertain participants to select an answer. Digital literacy was assessed using selected MTUAS subscales as a proxy. While it is practical to reduce respondent burden, this approach primarily measures technology use frequency rather than directly evaluating digital skills, confidence, or the independent ability to complete an electronic platform [40]. High internal consistency coefficients in several subscales may indicate item redundancy, supporting future validation of a shorter European Portuguese MHQ [43,50,51].

Finally, in the Portuguese context, this work provides a valuable tool for patient-reported outcomes in hand and wrist conditions. While other instruments are available, the MHQ offers a more comprehensive evaluation, uniquely assessing each hand separately and including domains like appearance and satisfaction [52,53]. The European Portuguese MHQ is a relevant and reliable instrument with adequate measurement properties for both clinical practice and research. The electronic version demonstrated superior precision and user preference. However, maintaining the paper format as a viable alternative is essential to ensure equitable access for patients with digital barriers. Due to the overall low absolute reliability between formats, once a patient-preferred format is adopted, it should be used consistently for longitudinal patient monitoring. Future research should assess the instrument in larger and more diverse samples, particularly regarding structural validity, external validity, and the performance of the electronic format in routine clinical and remote-care settings.

## Data Availability

The data that support the findings of this study are not openly available due to patient privacy and confidentiality.

## Appendix

Multimedia Appendix 1 This video file is a demonstration of the electronic version of the European Portuguese Michigan Hand Outcomes Questionnaire (MHQ). It shows the entire user process, from entering an ID and providing consent to answering the questions on the web-based platform. After completion, the video demonstrates the system's ability to provide instant, real-time scoring and an automatically generated, personalized report. This report, created with the assistance of an AI tool (ChatGPT-4o), summarizes the patient's results and compares them to normative data.

## Abbreviations

ADL: activities of daily living
COSMIN: Consensus-based Standards for the selection of health Measurement Instruments
GDPR: General Data Protection Regulation
ICC: intraclass correlation coefficient
ICHOM: International Consortium for Health Outcomes Measurement
ISPOR: International Society for Pharmacoeconomics and Outcomes Research
LoA: limits of agreement
MCID: minimal clinically important difference
MHQ: Michigan Hand Outcomes Questionnaire
MTUAS: Media and Technology Usage and Attitudes Scale
OMERACT: Outcome Measures in Rheumatology
PMR: physical medicine and rehabilitation
PROM: patient-reported outcome measure
SEM: standard error of measurement
STROBE: Strengthening the Reporting of Observational Studies in Epidemiology
SUS: System Usability Scale
